# Real-time cine and myocardial perfusion with treadmill exercise stress cardiovascular magnetic resonance in patients referred for stress SPECT

**DOI:** 10.1186/1532-429X-12-41

**Published:** 2010-07-12

**Authors:** Subha V Raman, Jennifer A Dickerson, Mihaela Jekic, Eric L Foster, Michael L Pennell, Beth McCarthy, Orlando P Simonetti

**Affiliations:** 1 Division of Cardiovascular Medicine, The Ohio State University, Columbus, Ohio, USA; 2 Dorothy M. Davis Heart and Lung Research Institute, The Ohio State University, Columbus, Ohio, USA; 3 Department of Biostatistics, The Ohio State University, Columbus, Ohio, USA; 4 Department of Radiology, The Ohio State University, Columbus, Ohio, USA

## Abstract

**Background:**

To date, stress cardiovascular magnetic resonance (CMR) has relied on pharmacologic agents, and therefore lacked the physiologic information available only with exercise stress.

**Methods:**

43 patients age 25 to 81 years underwent a treadmill stress test incorporating both Tc99m SPECT and CMR. After rest Tc99m SPECT imaging, patients underwent resting cine CMR. Patients then underwent in-room exercise stress using a partially modified treadmill. 12-lead ECG monitoring was performed throughout. At peak stress, Tc99m was injected and patients rapidly returned to their prior position in the magnet for post-exercise cine and perfusion imaging. The patient table was pulled out of the magnet for recovery monitoring. The patient was sent back into the magnet for recovery cine and resting perfusion followed by delayed post-gadolinium imaging. Post-CMR, patients went to the adjacent SPECT lab to complete stress nuclear imaging. Each modality's images were reviewed blinded to the other's results.

**Results:**

Patients completed on average 9.3 ± 2.4 min of the Bruce protocol. Stress cine CMR was completed in 68 ± 14 sec following termination of exercise, and stress perfusion CMR was completed in 88 ± 8 sec. Agreement between SPECT and CMR was moderate (κ = 0.58). Accuracy in eight patients who underwent coronary angiography was 7/8 for CMR and 5/8 for SPECT (p = 0.625). Follow-up at 6 months indicated freedom from cardiovascular events in 29/29 CMR-negative and 33/34 SPECT-negative patients.

**Conclusions:**

Exercise stress CMR including wall motion and perfusion is feasible in patients with suspected ischemic heart disease. Larger clinical trials are warranted based on the promising results of this pilot study to allow comparative effectiveness studies of this stress imaging system vs. other stress imaging modalities.

## Background

Treadmill exercise stress testing combined with nuclear or echocardiographic imaging forms a cornerstone in detection, prognostic evaluation and decision-making in patients with a broad spectrum of cardiovascular diseases, particularly atherosclerotic heart disease[1]. Exercise stress imaging studies provide information regarding location and extent of disease, with greater diagnostic accuracy than exercise ECG alone[1-4]. Despite widespread use[5,6], these modalities have limitations inherent to image acquisition technique that can affect accuracy. Obesity, prior surgery, or lung disease may limit stress echocardiography in some patients, and visualization of the posterolateral apex may be challenging in patients even with good acoustic windows[7]. Nuclear scintigraphic imaging with, most commonly, single photon emission computed tomography (SPECT) involves radiation exposure[8], is time-consuming for patients, and yields images with relatively low spatial resolution that may be further affected by photon scatter and breast or enteric attenuation artifact [9].

Cardiovascular magnetic resonance (CMR) requires no "acoustic window" and can freely visualize any plane in virtually any patient that can fit in the scanner - including those weighing over 400 lb in current wide-bore systems. Further, its higher spatial resolution affords demonstration of subendocardial perfusion defects that may not be apparent with other modalities [10-12]. To date, however, stress testing with CMR is almost exclusively performed with pharmacologic stress for several reasons: (i) standard exercise equipment is incompatible with MRI, (ii) CMR can be difficult under post-exercise conditions of high heart rate and rapid breathing, and (iii) the ECG signal is adversely affected by the magnetic field of the MRI system. Exercise stress offers a direct link between exertional symptoms and ischemia [13], in addition to information on functional capacity, blood pressure response and arrhythmias [14]. A supine bicycle ergometer that allows exercise imaging inside a closed-bore magnet has been commercially available for several years (Lode BV, The Netherlands). However, pedaling while supine is uncomfortable, atypical compared to patients' usual exertion and can be limited by leg fatigue. Knee-to-bore clearance while cycling is limited by patient height and magnet bore diameter, and the ECG signal is significantly distorted while the patient is inside or too near the MRI magnet [15].

The Bruce Treadmill Test, first published in 1963 [16], is the most commonly used exercise test protocol in the US [17,18] and has been shown to have high diagnostic and prognostic value [2,19]. Certain parameters such as inability to complete 6 minutes of the Bruce treadmill protocol [20] and inability to reach 85% of age-predicted maximum heart-rate indicate significant risk of coronary events [21], adding to the prognostic value of an exercise stress imaging test. While upright treadmill exercise is the physiologically preferred method of cardiovascular stress testing, it presents significant challenges with CMR. Standard treadmills are made from ferromagnetic components and powered by electromagnetic motors, preventing their safe use in close proximity to any MR magnet.

Rerkpattanapipat and colleagues showed feasibility of treadmill stress just outside the MR scanner room and post-exercise breathhold-cine imaging [22]. Their protocol required patients to walk from outside the room to the scanner table - patients with cardiorespiratory limitations may find this difficult to accomplish in a timely fashion, particularly after completing maximal exercise stress. Delays in completing stress imaging within 60-90 seconds of peak exercise reduce sensitivity in detecting ischemia due to recovery of ischemic segments[23,24]. To minimize the time between exercise and imaging, our group has shown feasibility of bringing a modified treadmill into the scanner room and incorporating both real-time nonbreathhold cine and first-pass myocardial perfusion acquisitions in the imaging protocol[25]. In the present study, we performed exercise stress CMR with our previously-developed in-room wall motion and perfusion imaging protocol in a cohort of patients referred for stress nuclear SPECT examination.

## Methods

Ambulatory patients scheduled for treadmill stress with single photon emission computed tomography (SPECT) imaging to evaluate known or suspected ischemic heart disease were screened for enrollment. Excluded were any patients with contraindications to magnetic resonance, such as pacemaker, cardiac defibrillator, cerebral aneurysm clip, ferromagnetic foreign body, or severe claustrophobia. Patients with significant renal insufficiency (estimated glomerular filtration rate from serum creatinine level within 30 days of enrollment ≤ 30 mL/min/1.73 m^2^) were also excluded. All patients provided written informed consent to participate in this Institutional Review Board-approved protocol.

### Imaging and exercise stress protocol

**Figure 1:**
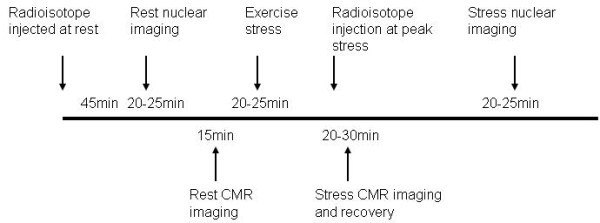
**Study Protocol**. Patients referred for clinically-indicated treadmill SPECT examination were enrolled in a combined protocol that allowed rest and post-exercise CMR in combination with the SPECT protocol with a single stress procedure.

A hybrid imaging protocol was employed so that both SPECT and CMR imaging could be completed for the same treadmill exercise procedure (Figure 1). Patients first underwent rest SPECT imaging using 9 mCi of 99m-Tc sestamibi using a standard gamma camera (General Electric MyoSIGHT, Milwaukee, WI). Then the patients were brought to the CMR room where a 1.5 Tesla scanner (Siemens Avanto, Erlangen, Germany) and 32-channel phased array coil (Rapid MRI, Columbus, OH) were used for all studies. Initial patient positioning and localization were done with two deflatable cushions - one placed under the head and shoulders and another below the calves and feet; thus, molds were created by deflating each cushion via in-room suction that allowed the patient to easily return to the same position on the scanner table post-exercise. This procedure enabled all slice positioning to be performed prior to exercise. Resting cine was performed using a non-triggered real-time steady-state free precession (SSFP) acquisition with TSENSE acceleration factor of 3 with the following typical scan parameters: TR 2.2 ms, TE 1.0 ms, flip angle (FA) 58°, receiver bandwidth (BW) 1360 Hz/pixel, temporal resolution 62.4 ms, slice thickness 8 mm, in-plane spatial resolution 3 × 3 mm. Cine acquisitions included 5 short-axis and 3 long-axis slices, with an acquisition window of 2 seconds for each slice. Cine and first-pass perfusion acquisitions were queued for immediate execution post-exercise. The table was then brought out of the scanner where the patient was connected to a 12-lead electrocardiography system (CardioSoft, GE Healthcare) for supine resting ECG. After standing on the treadmill and recording standing ECG at rest, exercise was performed using the Bruce treadmill stress protocol with a heart rate goal of 90% of maximum age-predicted heart rate (MPHR)[16]. Throughout stress, vital signs were recorded and a supervising physician and nurse evaluated the patient for symptoms and signs of distress. To insure adequate extraction time, the stress 99m-Tc sestamibi isotope dose of 31 mCi was injected approximately 90 seconds before terminating exercise. Standard criteria for termination prior to achieving target heart rate were observed including: patient request, significant arrhythmia, fall in systolic blood pressure > 10 mmHg, or any ST-segment elevation.

For post-exercise CMR imaging, the patient was quickly returned to the MR scanner table using the previously-fixed cushions to facilitate rapid, accurate re-positioning. The nurse attached the contrast injector tubing to the patient's intravenous line, and the patient was returned to the scanner isocenter for stress real-time cine imaging using the identical non-breathhold acquisition protocol used for rest cine imaging. Immediately upon completion of stress cine imaging (approximately 20 seconds), first-pass perfusion imaging was performed in three short-axis slices using a saturation recovery, hybrid gradient echo, echo-planar imaging sequence (TR/TE 5.6/1.1 ms, FA 25°, BW 1955 Hz/pixel, slice thickness 10 mm, in-plane spatial resolution 3 by 3 mm, echo train length of 4). TSENSE acceleration rate 2 resulted in a temporal resolution of 67.2 ms to acquire a typical 96 × 160 matrix. Preparation pulses (saturation and fat suppression) and saturation delay (30 ms) resulted in a total acquisition time of 127 ms per slice and simultaneous injection of 0.1 mmol/kg gadolinium-based contrast agent. The time elapsed from termination of exercise to completion of stress cine and stress perfusion imaging was recorded. Completion of stress perfusion was measured as the time to reach peak myocardial signal enhancement. After this scan was completed (40-50 seconds), the table was brought back out of the scanner and the 12-lead ECG system reconnected while the patient remained supine on the table for recovery monitoring for 4-6 minutes. After sufficient recovery to near resting heart rate and blood pressure, the patient was returned to the scanner's isocenter for recovery cine imaging. Rest perfusion imaging with a second injection of 0.1 mmol/kg gadolinium contrast injection was completed approximately 10 minutes after stress perfusion, and late gadolinium-enhancement imaging (LGE) followed 5-10 minutes later with appropriate inversion time selection to null normal myocardium. LGE was performed using a single-heartbeat non-breathhold scan (TR/TE 2.5/1.2 ms, FA 50°, BW 790 Hz/pixel, slice thickness 8 mm, in-plane spatial resolution 2-3 mm) in 8 to 12 short-axis planes covering the left ventricle. Upon completing CMR image acquisition, the patient returned to the adjacent SPECT camera for acquisition of stress scintigraphic images.

### Data analysis

The results of each study, exercise stress SPECT and exercise stress CMR, were interpreted by separate investigators blinded to the results of the other modality's interpretation. The nuclear interpreter had access to the gated SPECT images as well as rest and stress SPECT perfusion images. The CMR interpreter had access to the rest/stress cine and perfusion images as well as LGE images. Both readers had access to the exercise data - duration of stress, symptoms, vital signs, and ECG tracings. Each study was rated in aggregate review of treadmill, ECG, and all imaging data as negative for ischemia, fixed abnormality, or positive for ischemia. In a separate analysis focused on ability of each component of the exercise stress CMR test to detect ischemia specifically, we recorded negative/positive/indeterminate ECG, negative/positive cine wall motion and negative/positive perfusion and LGE; scar without perfusion abnormality beyond scar was recorded as 'negative' perfusion/LGE.

### Outcomes

Clinical outcomes at 6-month follow-up were recorded by telephone interview, and interim events such as hospitalization for angina, myocardial infarction were documented via chart review. Decision to perform x-ray coronary angiography was deferred to each patient's referring physician who was provided with both SPECT and CMR results. In patients referred for coronary angiography, results were recorded as presence or absence of ≥ 70% stenosis in any segment of the left main, left anterior descending coronary artery (LAD), left circumflex coronary artery (LCx), or right coronary artery (RCA) and major side branches.

### Statistical analysis

Continuous variables are reported as mean ± standard deviation (SD). A weighted kappa was used to measure agreement between nuclear and CMR interpreters in diagnosis of negative, fixed, and positive and a 95% bootstrapped confidence interval was constructed using the bias-corrected percentile method[26]. In the subset of patients who underwent coronary angiography after exercise stress, accuracy in detecting stenosis ≥ 70% was recorded using angiography as the gold standard. McNemar's test was used to compare the percentage of cases that were accurately classified as positive or negative by each modality. Due to the small sample size, we computed an exact p-value using SAS PROC FREQ (SAS V9.1, SAS Inc., Cary, NC).

## Results

Of forty-six patients initially enrolled, three were excluded due to: 1-patient stepping off the treadmill and refusing to complete either nuclear or CMR exam, 2-history obtained after enrollment revealed complex congenital heart disease and 3-sestamibi not available at the time of the study. Characteristics of the remaining 43 patients age 25 to 81 years are summarized in Table 1. Primary indication for stress testing was chest pain - 25 (58%), assessment of known CAD - 9 (21%), dyspnea - 4 (9%), or abnormal ECG - 5 (12%). Treadmill exercise stress was terminated after 9.3 ± 2.4 minutes of the Bruce protocol for the following endpoints: achieving 90% MPHR - 17 (40%), chest pain - 2 (5%), dyspnea - 11 (25%), fatigue - 11 (25%), or musculoskeletal pain - 2 (5%). Resting HR averaged 78 ± 16 beats per minute (BPM), and peak HR averaged 156 ± 21 bpm. Patients were not instructed to withhold HR-lowering medications for the stress test, and 33 (77%) reported use of a beta-blocker or calcium channel blocker at enrollment.

**Table 1:** Patient Population (N = 43)

Patient Characteristics	
Age (years)	54 ± 12
Body mass index, kg/m^2^	27.7 ± 4.2
Male	27 (63%)
Hypertension	25 (58%)
Diabetes mellitus	6 (10%)
Hypercholesterolemia	27 (63%)
Current smoker	8 (19%)
Known coronary artery disease	19 (44%)

Summary patient characteristics.

**Figure 2:**
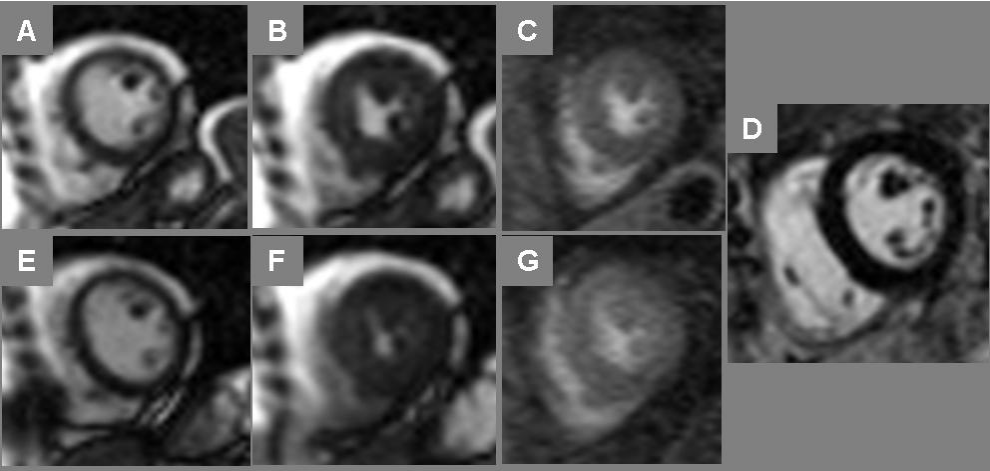
**Normal Treadmill Stress CMR**. End-diastolic (A, E) and end-systolic (B, F) frames of cine imaging at rest (top row) and immediately post-stress (bottom row) plus stress myocardial perfusion imaging (C, G) are shown in a 52 year-old postmenopausal female referred for stress SPECT to evaluate dyspnea; both stress modalities were negative for ischemia. In addition, late post-gadolinium enhancement (LGE) CMR imaging (D) showed no myocardial enhancement.

It took an average of 42.4 ± 5.2 s from the end of exercise to the start of imaging; this included the time required to get the patient on the table, strap on the receiver coil, connect the IV line, move the table to isocenter, and start the scan. Stress cine CMR imaging was completed by 68 ± 14 s following termination of exercise, and stress perfusion CMR imaging was completed by 88 ± 8 s, i.e. 20 seconds after completion of cine imaging. Absolute peak heart rate at end-exercise was 156 ± 21 bpm, which fell to 126 ± 19 bpm at time of cine imaging. As a percentage of maximum predicted heart rate, peak HR averaged 93 ± 9%, and HR at time of cine imaging averaged 74 ± 10%. In comparison, our prior study in healthy volunteers achieved a peak absolute HR of 177 ± 9 bpm that fell to 151 ± 18 bpm at imaging onset (corresponding to 98%MPHR at peak exercise falling to 84%MPHR at imaging onset). An illustration of normal wall motion and myocardial perfusion at rest and after treadmill stress is shown in Figure 2.

Technical problems occurred with perfusion imaging in 3 instances. In one, failure to unclamp the IV precluded administration of contrast to the patient during stress perfusion imaging acquisition. In a second, the in-room monitor was left on leading to RF interference and uninterpretable perfusion images. In a third, the ECG electrodes became dislodged in returning the patient to the scanner for stress imaging precluding triggering for perfusion imaging.

Overall procedural times were recorded as follows. Average time to complete rest imaging was 7:32 ± 3:17 (minutes:seconds). Completing the treadmill stress portion of the test - from the time the patient came out of the scanner to the time the patient re-entered the scanner for post-stress imaging - took 20:42 ± 5:39; stress imaging took on average 1:00 ± 0:25, and recovery time spanned 7:21 ± 2:38. The total procedure time averaged 44:52 ± 8:52.

**Figure 3:**
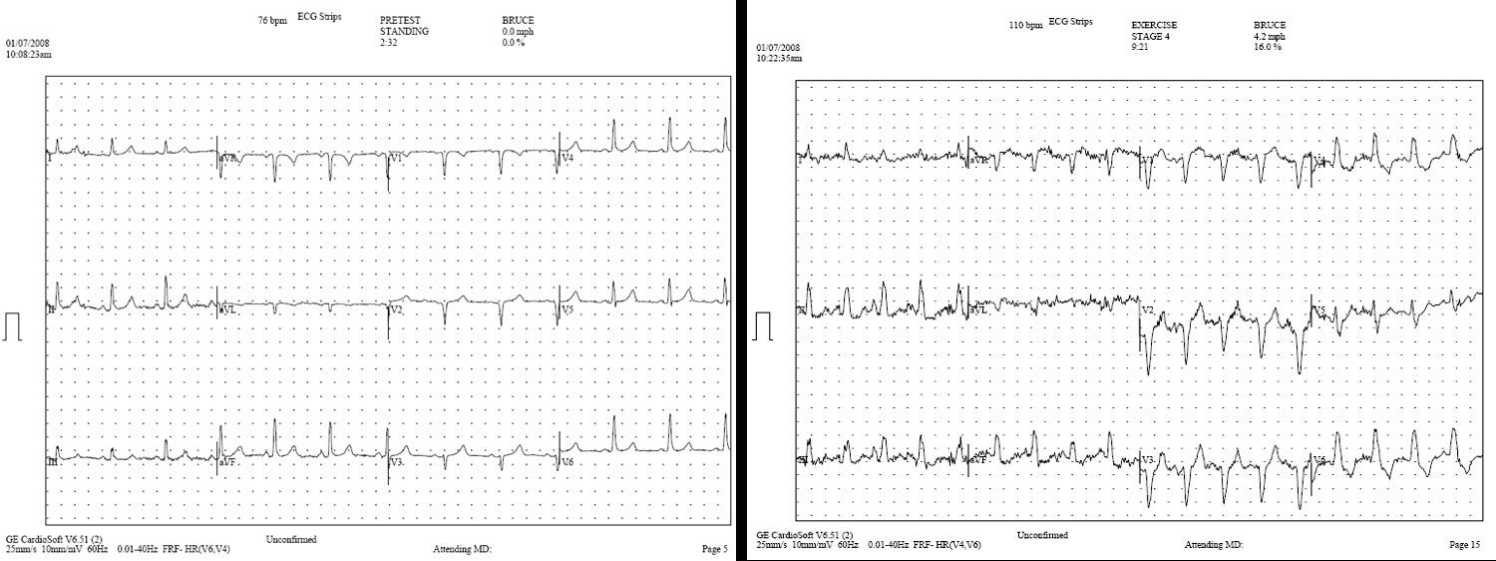
**Electrocardiography During Treadmill Stress CMR**. Rest (left) and stress (right) electrocardiography obtained in a 64 year-old male with exertional chest pain and remote anteroseptal myocardial infarction demonstrates exercise-induced left bundle branch block with reproduction of symptoms at stage 4 of the Bruce treadmill protocol.

**Figure 4:**
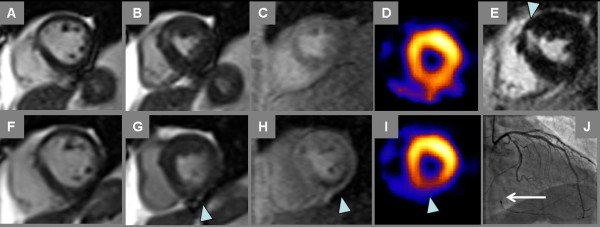
**Ischemia by Treadmill Stress CMR and SPECT**. Rest and stress CMR and SPECT images in the same patient of Fig. 3 both demonstrate myocardial ischemia, with corresponding obstructive coronary artery disease by angiography. Resting diastolic (A) and systolic (B) cine frames vs. comparable post-exercise cine frames (F, G) show stress-induced inferior wall contractile dysfunction (G, arrowhead). Inferior ischemia is also demonstrated by CMR perfusion imaging (C-rest perfusion vs. H-stress perfusion, arrowhead). Prior MI in the anteroseptum can be seen on late post-gadolinium imaging (E); note some fatty replacement in the infarct region evident as bright intramyocardial signal on noncontrast gradient echo cine frame in panel B. Rest Tc-99 m perfusion SPECT (D) suggests normal perfusion, though somewhat obscured by adjacent bowel uptake; stress Tc-99m perfusion SPECT shows inferior wall defect (I, arrowhead). The patient went on to invasive angiography that showed an occluded right coronary artery (J, arrow) with some left-to-right collateral flow.

**Figure 5:**
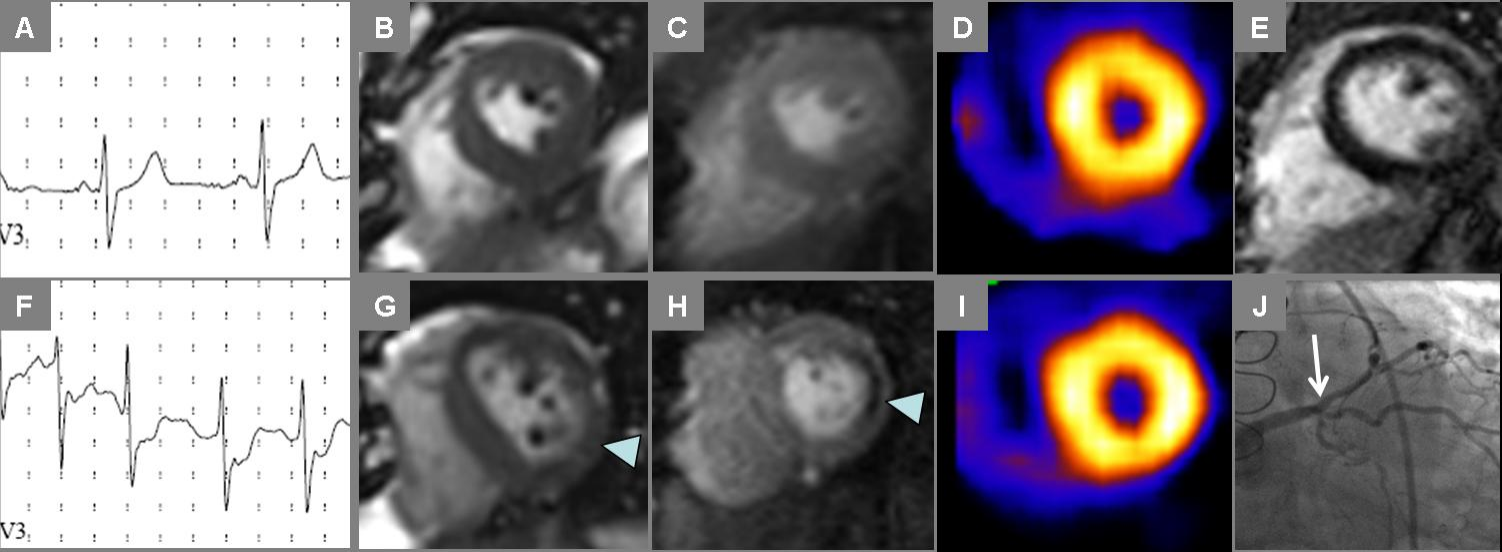
**Ischemia by Treadmill Stress CMR Not Evident by SPECT**. Rest and stress images show ischemia by CMR not evident by SPECT in a 56 year-old male with known coronary artery disease was referred for stress testing to evaluate abnormal stress ECG done prior to starting a supervised exercise program. Exercise-induced ischemia is evident by ST depression on electrocardiography (A-rest, F-stress), lateral wall motion abnormality on end-systolic frames from cine CMR (B-rest, G-stress) and lateral perfusion abnormality on first-pass contrast enhanced CMR (C-rest, H-stress). No myocardial infarct scar was seen by LGE CMR (E). SPECT images obtained during the same stress examination suggest normal myocardial perfusion (D-rest, I-stress). Invasive angiography (J) identified high-grade ostial stenosis of a large ramus intermedius coronary artery leading to percutaneous coronary intervention.

**Figure 6:**
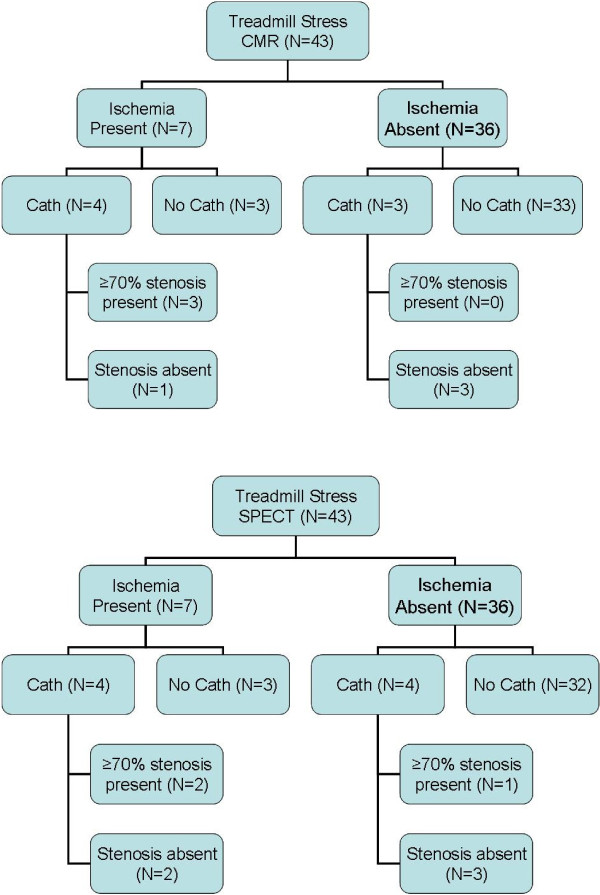
**Summary Findings of Treadmill Stress CMR, SPECT and Coronary Angiography**. Cardiac catheterization with x-ray coronary angiography (Cath) was performed in a subset of patients; angiographic results are shown in the context of CMR (a) or SPECT (b) indicating presence vs. absence of myocardial ischemia.

Test results and 6-month follow-up for all patients are listed in Table 2. Ten patients had ischemia by either stress modality, and 2 patients had fixed defects i.e. infarction without ischemia by both tests; agreement between MRI and SPECT was moderate (κ = 0.58, 95% CI 0.30 - 0.80). Of five patients with ischemia by both tests, all 5 went on to coronary angiography that showed ≥ 70% stenosis requiring revascularization in a coronary artery or bypass graft supplying the region of ischemia (Figures 3 and 4, Additional File 1). Of two patients with fixed defects, both had suffered prior MIs by clinical history and had undergone prior coronary revascularization. Additionally, CMR identified nontransmural infarct scar in 4 patients deemed to have normal stress SPECT exams. In 2 instances where stress SPECT suggested ischemia but stress CMR was negative, one patient went on to invasive coronary angiography and another underwent CT coronary angiography: both had angiographically-normal coronary arteries. Two patients with ischemia by stress CMR not seen by stress SPECT underwent invasive angiography: one was a male with ≥ 70% stenosis requiring revascularization (Figure 5), and the other was a female without epicardial stenosis in whom diffuse subendocardial ischemia was thought to represent microvascular disease. Summary outcomes of CMR, SPECT and angiography are presented in Figure 6.

**Table 2:** Individual Results

**Subject No**.	CMR Result	SPECT Result	Angiography	Outcome
1	1	1	0	1
2	1	1	0	1
3	1	3	1	1
4	3	3	2	1
5	1	1	0	CEA
6	3	3	0	2
7	1	1	1	1
8	2	2	0	1
9	2	1	0	0
10	1	1	0	1
11	1	1	0	1
12	3	1	2	1
13	1	1	0	1
14	1	1	1	1
15	1	1	0	1
16	2	2	0	1
17	1	1	0	0
18	1	1	0	1
19	1	1	0	1
20	1	1	0	1
21	1	1	0	1
23	1	1	0	1
24	3	3	0	1
25	3	3	0	1
26	1	1	0	1
27	3	1	1	1
28	2	1	0	1
29	1	1	0	1
30	1	1	0	1
32	2	3	0	1
33	1	1	0	1
34	3	3	2	1
36	1	3	1	1
37	1	1	0	1
38	1	1	0	1
39	1	1	0	1
40	1	1	0	1
41	1	1	0	1
42	2	1	0	1
43	1	1	0	1
44	1	1	0	1
45	1	1	0	1
46	4	1	0	1

Imaging results and outcomes in study subjects. CMR results are coded as follows: 1 = negative; 2 = infarct present, no ischemia; 3 = ischemia present. SPECT results are: 1 = negative; 2 = fixed defect; 3 = ischemia present; 4 = indeterminate. Angiography: 0 = not performed; 1 = performed and no obstructive disease; 2 = performed and obstructive disease present. Outcomes at 6-months follow-up are: 0 = lost to follow-up; 1 = no event; 2 = cardiovascular hospitalization. CEA = carotid endarterectomy. Notes: Excluded were subjects 22 (who jumped off the treadmill prior to termination and refused study completion), 31 (complex congenital heart disease) and 35 (no sestamibi).

In analyzing individual components of the exercise CMR exam in the 40 cases where perfusion images were adequate, overall concordance between cine CMR and perfusion CMR was excellent: there were only 2 cases of discordant results. In one (Subject 24), perfusion indicated diffuse subendocardial ischemia but cine did not; interestingly, this patient had undergone coronary CTA 2 months prior to enrollment that indicated extensive coronary ectasia without obstruction. In the second (Subject 27), cine indicated ischemia (septal wall motion abnormality with stress) where perfusion did not; this patient notably had midmyocardial fibrosis most prominent in the septum.

Of 8 subjects who had invasive coronary angiography, stress CMR findings of present/absent ischemia were correct in 7 subjects (one false-positive) compared to 5 (2 false-positive and 1 false-negative) stress SPECT findings; this small subset precluded detection of a significant difference in classification accuracy (p = 0.63). With respect to the exercise ECG data in 8 patients who underwent angiography, stress ECG indicated ischemia in 1 patient in whom cine CMR, perfusion CMR and SPECT images were negative for ischemia. In three patients, ECG was indeterminate due to resting abnormalities; 2 of these underwent angiography, one of whom showed ischemia both cine and perfusion CMR as well as SPECT and the other whose CMR and SPECT data indicated no ischemia.

Six-month freedom from cardiovascular events was high for patients with normal findings by either modality: all 29 patients with a negative stress CMR and 33 of 34 patients with a negative stress SPECT suffered no documented myocardial ischemia, infarction or death at 6-month follow-up.

## Discussion

In this study using an in-room implementation for treadmill stress with CMR, we showed the feasibility of detecting ischemia in patients with known or suspected coronary artery disease. Agreement was moderate with SPECT, the modality that comprises the vast majority of stress imaging tests performed in the United States. Stress cine imaging was completed, on average, 68 seconds following termination of exercise; stress perfusion imaging followed cine and required an additional 20 seconds. Societal guidelines for exercise stress echocardiography state that imaging of cardiac function must be completed within two minutes, and preferably less than one minute after exercise. No similar guidelines exist for perfusion imaging, which is typically performed by nuclear scintigraphy that does not require image capture immediately post-stress. While this configuration with the treadmill positioned approximately 10 feet from the MR scanner table fell short of the one minute guideline for stress echocardiography, accuracy and prognostic value in this small cohort were encouraging. The number of patients who underwent catheter angiography was limited and we did not perform quantification or additional visual analysis of angiographic data; nonetheless, diagnosis of ischemia by exercise stress CMR corresponded to significant coronary stenoses, while negative results predicted absence of disease and event-free survival.

Compared to our prior work testing the identical partially-modified treadmill stress CMR system in younger cohort of volunteers[25], the patients requiring stress SPECT for known or suspected ischemic heart disease in this work were older (mean age 54 vs. 39 years) and took considerably longer to transfer from the treadmill in the corner of the room to the scanner for post-stress imaging (42 vs. 30 seconds on average in patients vs. volunteers, respectively). The drop in heart rate was slower post-exercise, though the %MPHR at onset of imaging was lower (74% vs. 84%) raising concern that ischemia detection may be inadequate without additional improvements to shorten the time to imaging in typical cardiac patients.

Schwitter *et al*. in a multicenter effort demonstrated that vasodilator stress perfusion CMR provided highly accurate detection compared with stress SPECT myocardial perfusion imaging[27]. How treadmill stress CMR would compare to pharmacologic stress CMR is unknown. There will remain a portion of patients who are unable to undergo treadmill stress that require inotropic or vasodilator stress; for those who can exercise, however, treadmill stress CMR may be a new option for physiologic cardiac stress imaging. The physiologic information yielded by observing a patient's response to standardized exercise stress - exercise capacity, rhythm and heart rate response/recovery, and reproduction of exertional symptoms - can now be coupled with CMR's high-resolution cardiac wall motion, perfusion and scar imaging. Patients could complete a treadmill stress CMR visit well within 1 hour, compared to a typical 2-4 hour commitment for exercise SPECT.

Subclinical infarct scar adversely influences prognosis even in the absence of reported MI history[28]. SPECT tends to miss these infarcts, especially if nontransmural, due to lower spatial resolution, missing an opportunity to make a diagnosis that has both prognostic and therapeutic implications. Other advantages that may make exercise CMR a useful modality in specific patient populations include reliable imaging of the right heart, simultaneous acquisition of flow data and imaging in any plane, all of which may be particularly helpful in evaluating patients with congenital heart disease.

Despite encouraging results, any enthusiasm for this new modality of stress cardiac imaging must be tempered by several limitations. First, the time to image acquisition exceeded the limit advocated in stress echo guidelines, which is relevant since treadmill stress CMR requires image acquisition after completion of exercise just like stress echo. Technical advances that allow placement of the treadmill immediately next to the scanner table would shorten the time to image acquisition, potentially to within the target of 1 minute. While we expect that shortening the delay will have a positive impact on sensitivity in detecting obstructive coronary disease with CMR, further data is required to establish the magnitude of this effect. CMR remains impossible in patients with ferromagnetic foreign bodies or non-MR compatible implants in whom myocardial SPECT and echocardiography remain feasible. While renal insufficiency may preclude gadolinium-based contrast administration, cine CMR may be done without contrast. Further analyses in larger patient cohorts are needed to determine the utility of stress CMR with cine imaging alone without LGE or perfusion imaging. The established diagnostic and prognostic value of dobutamine stress CMR without gadolinium contrast motivates further study to determine whether or not noncontrast treadmill stress CMR offers comparable utility[29].

## Conclusions

In conclusion, treadmill exercise stress CMR with wall motion and perfusion imaging in the MR scanner room is feasible in patients referred for stress testing, and shows moderate agreement with stress SPECT. Further studies that leverage technical advances to reduce the time between treadmill exercise and completion of post-exercise imaging are needed before widespread clinical implementation.

## Competing interests

SVR, ELF, and OPS have applied for a patent on the MRI-compatible treadmill and have ownership interest in EXCMR, Ltd., a company commercializing this device.

SVR and OPS receive research support from Siemens Medical Solutions, Inc.

## Authors' contributions

SVR and OPS conceived of the study, participated in its design and coordination, and drafted the manuscript. JAD, BM, MJ, and ELF carried out the experiments. MJ, ELF, and OPS designed and constructed the partially-modified treadmill stress system used in this work. JAD, MJ, and SVR analyzed the data. MLP participated in the design of the study and the statistical analysis. All authors read and approved the final manuscript.

## Supplementary Material

Additional file 1 **Stress SPECT, CMR and Angiography**. Cine images are shown corresponding to the still images of Figure 4. Click here for file
