# Supplementary figures and images for: Real-time cine and myocardial perfusion with treadmill exercise stress cardiovascular magnetic resonance in patients referred for stress SPECT
Source: J Cardiovasc Magn Reson. 2010 Jul 12;12(1):41. doi: 10.1186/1532-429X-12-41 (PMC2908608; doi:10.1186/1532-429X-12-41)

## Slide 1
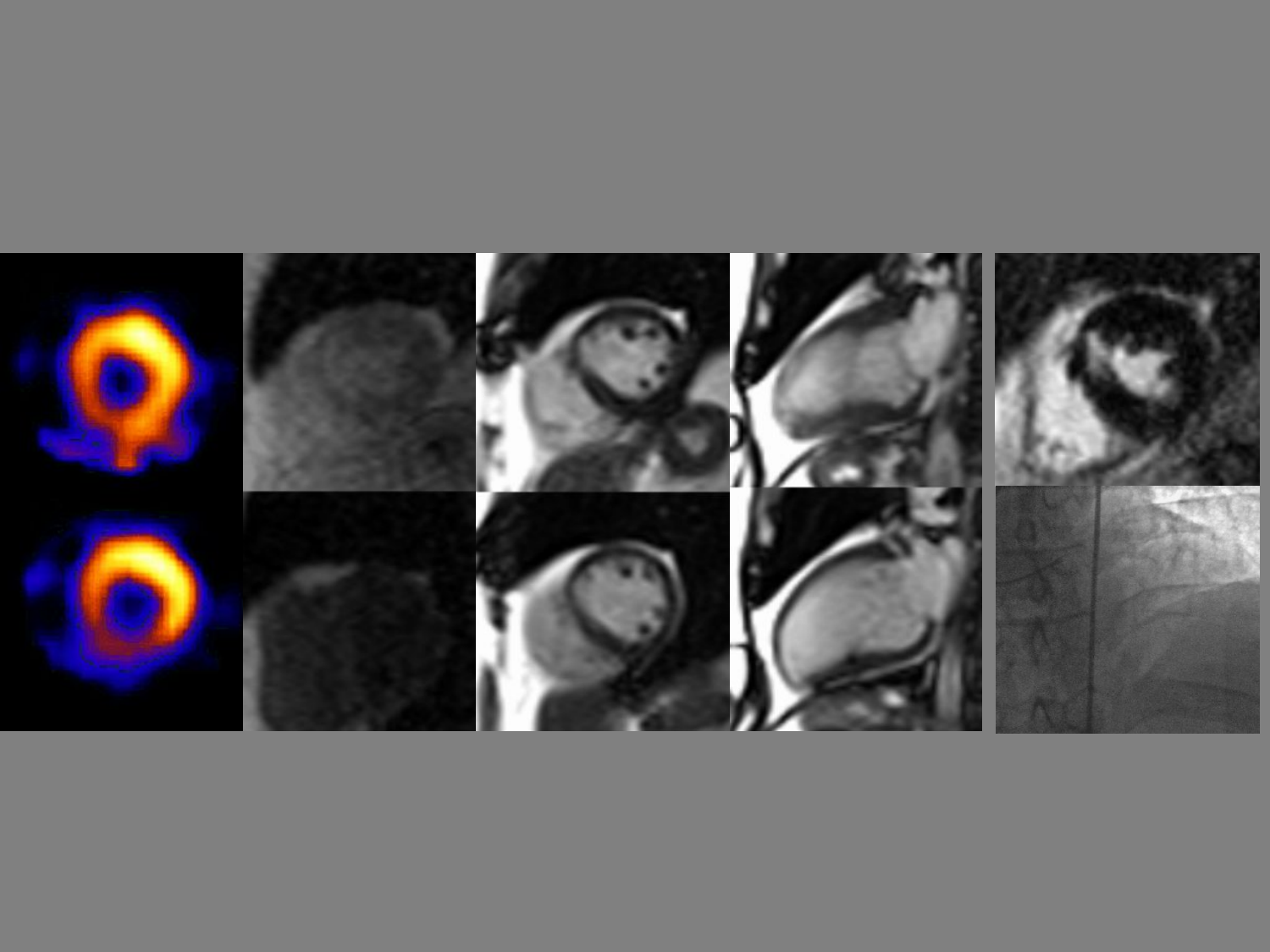

Supplement: Additional file 1 — Stress SPECT, CMR and Angiography. Cine images are shown corresponding to the still images of Figure 4. [file 1532-429X-12-41-S1.PPT]
